# Updated carrier rates for c.35delG (*GJB2*) associated with hearing loss in Russia and common c.35delG haplotypes in Siberia

**DOI:** 10.1186/s12881-018-0650-5

**Published:** 2018-08-07

**Authors:** Marina V. Zytsar, Nikolay A. Barashkov, Marita S. Bady-Khoo, Olga A. Shubina-Olejnik, Nina G. Danilenko, Alexander A. Bondar, Igor V. Morozov, Aisen V. Solovyev, Valeriia Yu. Danilchenko, Vladimir N. Maximov, Olga L. Posukh

**Affiliations:** 1 Federal Research Center Institute of Cytology and Genetics SB RAS, Novosibirsk, Russia; 2 Novosibirsk State University, Novosibirsk, Russia; 3 Yakut Scientific Centre of Complex Medical Problems, Yakutsk, Russia; 4 M.K. Ammosov North-Eastern Federal University, Yakutsk, Russia; 5 Scientific Research Institute of Medical-Social Problems and Management of the Republic of Tuva, Kyzyl, Russia; 6 Institute of Genetics and Cytology, National Academy of Sciences, Minsk, Belarus; 7 Institute of Chemical Biology and Fundamental Medicine SB RAS, Novosibirsk, Russia

**Keywords:** Deafness, *GJB2*, c.35delG, Haplotypes, Siberia

## Abstract

**Background:**

Mutations in *GJB2* gene are a major causes of deafness and their spectrum and prevalence are specific for various populations. The well-known mutation c.35delG is more frequent in populations of Caucasian origin. Data on the c.35delG prevalence in Russia are mainly restricted to the European part of this country. We aimed to estimate the carrier frequency of c.35delG in Western Siberia and thereby update current data on the c.35delG prevalence in Russia. According to a generally accepted hypothesis, c.35delG originated from a common ancestor in the Middle East or the Mediterranean ~ 10,000–14,000 years ago and spread throughout Europe with Neolithic migrations. To test the c.35delG common origin hypothesis, we have reconstructed haplotypes bearing c.35delG and evaluated the approximate age of c.35delG in Siberia.

**Methods:**

The carrier frequency of c.35delG was estimated in 122 unrelated hearing individuals living in Western Siberia. For reconstruction of haplotypes bearing c.35delG, polymorphic D13S141, D13S175, D13S1853 flanking the *GJB2* gene, and intragenic rs3751385 were genotyped in deaf patients homozygous for c.35delG (*n* = 24) and in unrelated healthy individuals negative for c.35delG (*n* = 67) living in Siberia.

**Results:**

We present updated carrier rates for c.35delG in Russia complemented by new data on c.35delG carrier frequency in Russians living in Western Siberia (4.1%). Two common D13S141-c.35delG-D13S175-D13S1853 haplotypes, 126-c.35delG-105-202 and 124-c.35delG-105-202, were reconstructed in the c.35delG homozygotes from Siberia. Moreover, identical allelic composition of the two most frequent c.35delG haplotypes restricted by D13S141 and D13S175 was established in geographically remote regions: Siberia and Volga-Ural region (Russia) and Belarus (Eastern Europe).

**Conclusions:**

Distribution of the c.35delG carrier frequency in Russia is characterized by pronounced ethno-geographic specificity with a downward trend from west to east. Comparative analysis of the c.35delG haplotypes supports a common origin of c.35delG in some regions of Russia (Volga-Ural region and Siberia) and in Eastern Europe (Belarus). A rough estimation of the c.35delG age in Siberia (about 4800 to 8100 years ago) probably reflects the early formation stages of the modern European population (including the European part of the contemporary territory of Russia) since the settlement of Siberia by Russians started only at the end of sixteenth century.

**Electronic supplementary material:**

The online version of this article (10.1186/s12881-018-0650-5) contains supplementary material, which is available to authorized users.

## Background

Pathogenic variants in the *GJB2* gene (MIM 121011, chromosome 13q11q12) are the most common causes of autosomal recessive non-syndromic hearing loss in various populations. Over 300 pathogenic variations in *GJB2* have been reported in the Human Gene Mutation Database [1]. Many of them have high ethno-geographic specificity in prevalence [2], which for certain ethnic groups is being attributed to a founder effect [3–11].

The recessive pathogenic *GJB2* variant c.35delG (p.Gly12Valfs*2) (NM_004004.5) is known to be prevalent in deaf patients of Caucasian origin [2, 12, 13]. Previous studies have revealed the c.35delG carrier frequency to be around 1 in 50 overall in Europe [12], reaching 1 in 31 in Southern Europe [14]. In meta-analysis of the data published up to 2008, mean carrier frequencies of c.35delG were found to be 1.89, 1.52, 0.64, 1, and 0.64% for European, American, Asian, Oceanic, and African populations, respectively [13]. The c.35delG is a deletion of one guanine (G) from a string of six (GGGGGG) in the *GJB2* coding sequence resulting in a frameshift and termination of the Cx26 protein sequence at amino acid 13 (p.Gly12Valfs*2). The occurrence of c.35delG as a possible “hot spot” caused by DNA polymerase “slippage” was previously assumed to be an explanation of the high prevalence of this pathogenic variant in the *GJB2* gene [5, 15, 16]. Nevertheless, convincing evidence emerged that the founder effect plays an important role in the prevalence and accumulation of c.35delG in populations of Caucasian origin since lower rates or absence of c.35delG are observed in other populations. According to a generally accepted hypothesis, c.35delG originated from a common ancestor in the Middle East or the Mediterranean approximately 10,000–14,000 years ago and spread throughout Europe with Neolithic migrations. Specific c.35delG prevalence and discovery of common STR- and SNP-haplotypes bearing the c.35delG mutation in Mediterranean, Middle Eastern, North-European populations, and in individuals of European origin in the USA support this hypothesis [14, 17–27]. Relevant data were also obtained for populations of the Volga-Ural region of Russia [28] and Belarus [29].

The c.35delG predominance in deaf patients was reported in several studies conducted in the European part of Russia [30–38]. In the ethnically heterogeneous population of Siberia, epidemiological and molecular genetic studies of hereditary deafness are currently limited to regions of the Altai Republic, the Tuva Republic (Southern Siberia), and the Sakha Republic (Yakutia, Eastern Siberia) [39–41]. The presence of c.35delG in a homozygous or compound-heterozygous state was the main cause of hereditary hearing loss in deaf Russian patients living in these regions in contrast to deaf patients belonging to Siberian indigenous peoples (Altaians, Tuvinians, Yakuts) who were negative for the c.35delG mutation [39–41].

This study presents an updated summary of published data on the c.35delG (p.Gly12Valfs*2) prevalence in Russia complemented by our original data on the c.35delG carrier frequency in Western Siberia. To test the c.35delG common origin hypothesis, we genotyped polymorphic markers flanking the *GJB2* gene and reconstructed haplotypes bearing c.35delG in deaf patients from Siberia homozygous for c.35delG.

## Methods

### Subjects

Twenty-four unrelated patients (mostly Russians) with congenital or early onset profound hearing loss living in several Siberian regions (Altai, Tuva, Yakutia) were previously found to be homozygous for c.35delG [39–41]. The carrier frequency of c.35delG was estimated in 122 unrelated normal hearing individuals (mostly Russians) from the Novosibirsk region (Western Siberia). Genotyping of three polymorphic short tandem repeat (STR) markers D13S141, D13S175, D13S1853 and an intragenic SNP (rs3751385) was performed in 24 unrelated deaf patients homozygous for c.35delG and in 67 unrelated healthy individuals from the Novosibirsk region (Western Siberia) who were negative for c.35delG.

### C.35delG screening and analysis of genetic markers

All primers and genotyping methods are summarized in Table 1. The c.35delG screening was performed according to [42]. Polymorphic STR markers flanking the *GJB2* gene: D13S141 (~ 39.2 kb centromeric to c.35delG), D13S175 and D13S1853 (~ 84.8 kb and ~ 277.1 kb telomeric to c.35delG, respectively), and intragenic SNP (rs3751385) locating ~ 0.7 kb centromeric from c.35delG were used to reconstruct haplotypes bearing c.35delG. These markers were used previously in relevant studies and were therefore chosen to keep compatibility and enable comparative analysis with already available data. Genotyping of D13S141, D13S175 and D13S1853 was performed in the SB RAS Genomics Core Facility (Institute of Chemical Biology and Fundamental Medicine SB RAS, Novosibirsk, Russia).

**Table 1:** Primer sequences for PCR, fragment analysis and Sanger sequencing

c.35delG and studied markers (localization, GRCh38.p12)^a^	Primer sequences	Methods of detection
c.35delG (*GJB2*) (13:20189547)	F: 5′-GGTGAGGTTGTGTAAGAGTTGG-3′ R: 5’-CTGGTGGAGTGTTTGTTCC*CAC-3’	PCR-mediated site-directed mutagenesis (PSDM) with use of *Bsc4 I*
D13S141^b^ (13:20150320–20,150,445)	F: 5’-GTCCTCCCGGCCTAGTCTTA-3’ (6-FAM) R: 5’-ACCACGGAGCAAAGAACAGA-3’	Fragment analysis (GeneScan 500 LIZ) on ABI 3130XL (Applied Biosystems)
D13S175^b^ (13:20274367–20,274,479)	F: 5’-TATTGGATACTTGAATCTGCTG-3’ (PET) R: 5’-TGCATCACCTCACATAGGTTA-3’	
D13S1853^b^ (13:20466607–20,466,800)	F: 5’- CAGACTGGCACAAACTTAACTG −3’ (6-FAM) R: 5’- TGTACATCTCTTCTTACATTCATGT − 3’	
rs3751385 (13:20188817)	F: 5’-GGCTGGTGAAGTGCAACG-3′ R: 5’-GTAAGCAAACAAACTTTTGAAGTAG-3’	PCR-RFLP analysis with use of *Nhe I*

^a^Localization was taken from the Ensembl Genome browser [53]; ^b^ - Specific primer sequences for PCR amplification of microsatellites D13S141, D13S175, and D13S1853 were obtained from the Ensembl genome browser and the NCBI Probe Database [53, 54], one from each primer pairs was labeled with the fluorescent dyes

### Statistical analysis

Haplotype frequencies were estimated from observed genotype data using Expectation–Maximization (EM) algorithm of the Arlequin 3.5.2.2 software [43]. Fisher’s exact test (significance level 0.05) was used to compare the allelic and haplotype distributions. Linkage disequilibrium between the marker alleles and c.35delG as well as the age of c.35delG were estimated as described previously [44, 45]. The linkage disequilibrium was calculated as

$$ \updelta =\left(\mathrm{Pd}-\mathrm{Pn}\right)/\left(1-\mathrm{Pn}\right), $$

where δ is the measure of linkage disequilibrium, Pd is the frequency of the marker allele among all chromosomes carrying c.35delG, and Pn is the frequency of the same allele among chromosomes without c.35delG. The age of c.35delG was estimated as

$\text{g} = \text{log} [1-\text{Q}/(1-\text{Pn})]/\text{log}(1-\ominus)$

where g is the number of generations from the moment of the c.35delG appearance to the present, Q is the share of chromosomes carrying c.35delG unlinked with the founder haplotype, Pn is the frequency of the allele included in founder haplotype in the population, and Ѳ is the recombinant fraction calculated from the physical distance of the markers from the c.35delG location on the assumption of 1 cM = 1000 kb.

## Results

### Carrier frequency of c.35delG in Russia

**Fig. 1:**
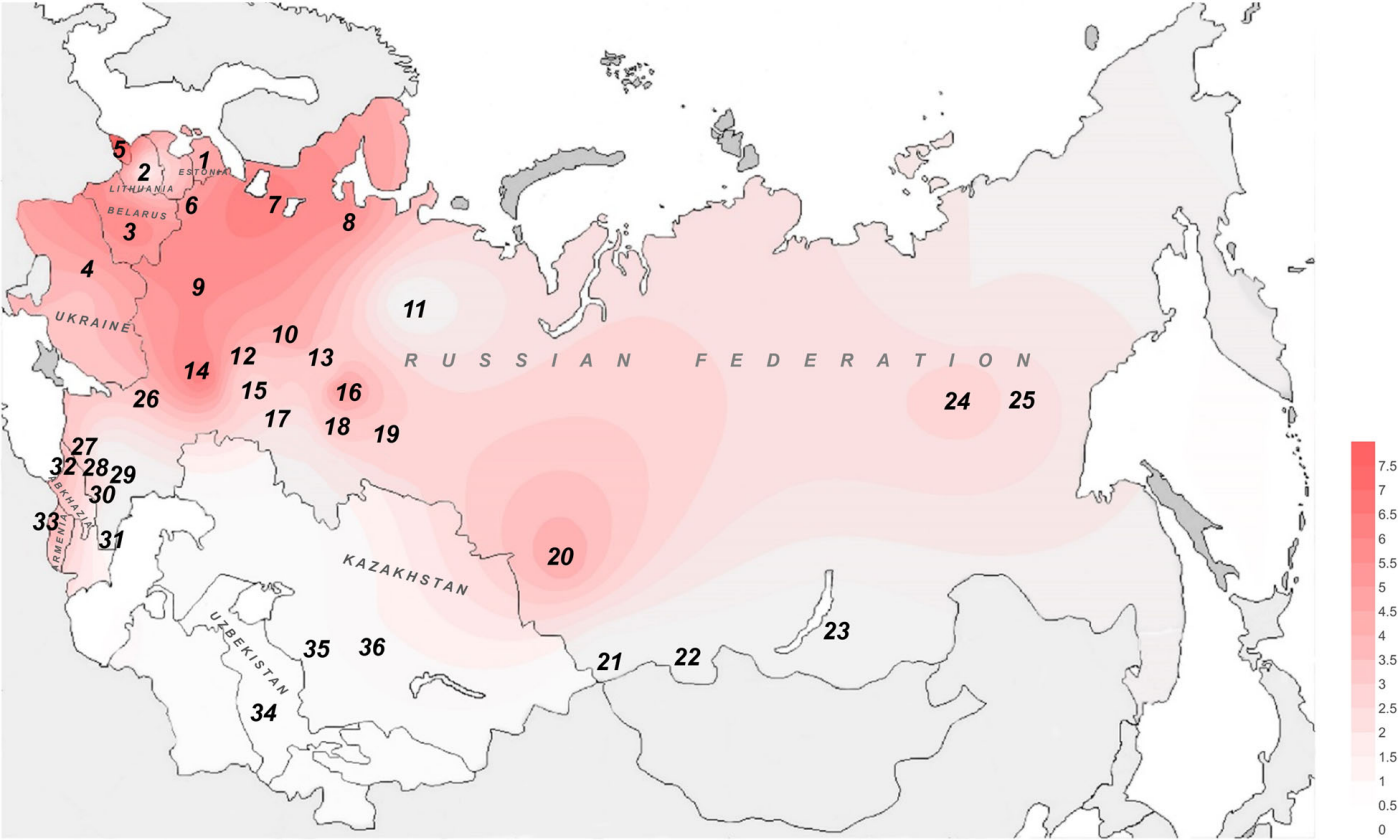
Distribution of the c.35delG carrier frequency on the territory of Russia and in some countries of the former Soviet Union. The c.35delG carrier frequencies (%) were obtained from all available data published up to 2018 (Additional file 1: Table S1). Codes from 1 to 36 indicate analyzed samples (region under study and/or ethnicity which were indicated in the original publications). In some cases, c.35delG carrier frequency was calculated by us from the data given in original publications. The maximum value of the c.35delG carrier frequency is used for the figure if there are several data sets for a region or an ethnically stratified sample. **Codes** ***1–4 – Northern and Eastern Europe***: ***1*** – Estonia (4.4–4.5%), ***2*** – Lithuania (1.0%), ***3*** – Belarus (3.4–6.2%), ***4*** – Ukraine (3.3–4.1%); ***5–8 - North-Western part of Russia***: ***5*** – residents of Kaliningrad and Kaliningradskaya Oblast’ (7.5%), ***6*** - residents of Pskov and Pskovskaya Oblast’ (2.0–4.7%), ***7*** - residents of St. Petersburg and Leningradskaya Oblast’ (3.3–5.9%), ***8*** - residents of Arkhangelsk and Arkhangelskaya Oblast’ (5.0%); ***9–10 - Central part of Russia***: ***9*** – residents of different regions (3.8–5.1%); ***10*** – Russians (Kirovskaya Oblast’) (3.8%); ***11–19 - Volga-Ural region of Russia***: ***11*** – Komi (0%), ***12*** - Mari (2.0–2.6%), ***13*** - Udmurts (0.5–3.7%), ***14*** – Mordvins (5.7–6.2%), ***15*** – Chuvashes (0–2.6%), ***16*** - Russians (5.0%), ***17*** - Tatars (1.0–2.6%), ***18*** - Bashkirs (0–3.6%), ***19*** – Russians (Ekaterinburg) (2.2%); ***20–25 – Siberia***: ***20*** - Russians (Novosibirsk, Western Siberia) (4.1%), ***21*** – Altaians (0%), ***22*** – Tuvinians (0%); ***23*** *-* Buryats (0%); ***24*** - Yakuts (0.4–1.0%), ***25*** - Russians (Yakutia, Eastern Siberia) (2.5%); ***26–31 - South-Western part of Russia (including North Caucasus)***: ***26*** - residents of Rostovskya Oblast’ (Russians) (2.9%), ***27*** – Cherkessians (1.3–2.0%), ***28*** – Karachays (0.3%), ***29*** - Ingush (0–2.0%), ***30*** - Chechens (0–0.7%), ***31***- Avars (0%); ***32–33 - South Caucasus: 32*** – Abkhazians (3.8%), ***33*** – Armenians (3.7%); ***34–36 - Central Asia***: ***34*** - Uzbeks (0%), ***35*** - Kazakhs (0.8%), ***36*** - Uighurs (0.9%)

Screening of c.35delG in unrelated hearing individuals (mostly Russians) living in the Novosibirsk region (Western Siberia) has revealed 5 out of 122 examined subjects to be c.35delG carriers (4.1%). These data supplement current information about the c.35delG prevalence in Russia. We have analyzed all available literature data (published up to 2018) on c.35delG carrier frequencies in Russia and in some countries of the former Soviet Union (USSR) which populations undoubtedly contributed to the contemporary population of Russia. The distribution of c.35delG carrier frequencies is presented in Fig. 1. High c.35delG frequencies are observed in the populations of north-western and central parts of Russia with downward trend from west to east.

### Common haplotypes associated with c.35delG in Siberia

**Fig. 2:**
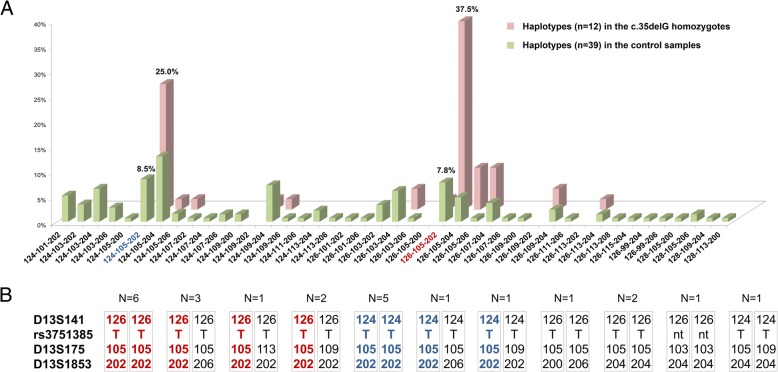
The D13S141-D13S175-D13S1853 haplotypes in studied samples. **a** Distribution of the D13S141-D13S175-D13S1853 haplotypes reconstructed by the Arlequin software (EM algorithm) in the c.35delG homozygotes (pink bars) and in the control samples (green bars) (see explanation in text). n – number of haplotypes. **b** The D13S141-rs3751385-c.35delG-D13S175-D13S1853 haplotypes found in the c.35delG homozygotes. N - number of individuals. The most frequent haplotypes are highlighted in color: 126-T-105-202 – by red, 124-T-105-202 – by blue; nt - not tested

Certain polymorphic STR markers flanking *GJB2* are traditionally used for c.35delG haplotype analysis: centromeric D13S141 (~ 39.1 kb from c.35delG), telomeric D13S175 (~ 84.8 kb from c.35delG) and distal telomeric D13S143 (~ 1.5 Mb from c.35delG) [17, 19–29]. Since c.35delG is presumably a very old mutation, a common ancestral haplotype could be observed in even a closest chromosomal locus, therefore instead of the more distal D13S143, we chose the marker D13S1853 that was closer to c.35delG (~ 277.1 kb telomeric), making the haplotype region (D13S141-c.35delG-D13S175-D13S1853) span over ~ 316 kb. Results of D13S141, D13S175, D13S1853 and rs3751385 genotyping in the c.35delG homozygotes and in control individuals from Siberia are summarized in Table 2. Significant differences in allele frequencies of markers D13S141, D13S175, D13S1853 are observed between the c.35delG homozygotes and control subjects (Table 2). Allele T of rs3751385 was only identified in the c.35delG homozygotes showing significant differences (*p* <  10^− 21^) between patients and control samples. A strong association of allele T (rs3751385) with c.35delG was also shown in previous studies [18, 22–25, 27, 29]. Twelve and thirty-nine D13S141-D13S175-D13S1853 haplotypes were reconstructed for c.35delG homozygotes and for control samples from Siberia, respectively (Fig. 2A). Two haplotypes, 126–105-202 (37.5%) and 124–105-202 (25.0%), were the most common (52.5% in total) among chromosomes bearing c.35delG in contrast with 7.8% for 126–105-202 and 8.5% for 124–105-202 among wild-type chromosomes (p <  10^− 2^–10^− 5^). Based on observed linkage disequilibrium for D13S175 and D13S1853 alleles (Table 2), we have roughly estimated the age of c.35delG in Siberia as ~ 4800 years (D13S1853) or ~ 8100 years (D13S175). It should be noted that an accurate calculation of the c.35delG age is difficult because of many uncertainties, first of all, unknown true recombination frequency of this chromosomal region [18, 22].

**Table 2:** Allele frequencies of D13S141, D13S175, D13S1853, and rs3751385 in the c.35delG homozygotes and in the control samples (individuals without c.35delG)

Markers (location from c.35delG)	Allele^a^	Homozygotes for c.35delG (*n* = 24)					Control samples (*n* = 67)	
		Number of alleles	Allele frequency	χ^2^	p	δ	Number of alleles	Allele frequency
D13S141 (~ 39.2 kb centromeric)	120	0	0.0000	–	–	0.0000	0	0.0000
	122	0	0.0000	–	–	0.0000	0	0.0000
	124	16	0.3333	7.8	**0.0025**	−0.5953	78	0.5821
	126	32	0.6667	11	**0.0006**	**0.4618**	51	0.3806
	128	0	0.0000	0.71	0.2121	−0.0387	5	0.0373
	Total	48					134	
rs3751385 (intragenic, ~ 0.7 kb centromeric)	C	0	0.0000	76	**< 10** ^**−21**^	−5.1652	62	0.8378
	T	46	1.0000	76	**< 10** ^**−21**^	**1.0000**	12	0.1622
	Total	46					74	
D13S175 (~ 84.8 kb telomeric)	99	0	0.0000	0.002	0.5410	−0.0151	2	0.0149
	101	0	0.0000	2.1	0.0591	−0.0720	9	0.0672
	103	2	0.0417	7.3	**0.0016**	−0.2467	31	0.2313
	105	41	0.8542	28	**< 10** ^**−7**^	**0.7588**	53	0.3955
	107	0	0.0000	2.5	**0.0427**	−0.0806	10	0.0746
	109	4	0.0833	0.45	0.2570	−0.0589	18	0.1343
	111	0	0.0000	0.002	0.5410	−0.0151	2	0.0149
	113	1	0.0208	0.46	0.2616	−0.0413	8	0.0597
	115	0	0.0000	0.29	0.7363	−0.0076	1	0.0075
	Total	48					134	
D13S1853 (~ 277.1 kb telomeric)	200	1	0.0208	0.092	0.4074	−0.0251	6	0.0448
	202	34	0.7083	23	**< 10** ^**−6**^	**0.5842**	40	0.2985
	204	8	0.1667	16	**< 10** ^**−4**^	−0.6920	68	0.5075
	206	5	0.1042	0.17	0.3497	−0.0439	19	0.1418
	208	0	0.0000	0.29	0.7363	−0.0076	1	0.0075
	Total	48					134	

^**a**^ - Designation of the STR allele corresponds to its size in nucleotides; δ - measure of linkage disequilibrium; the maximum indices of linkage disequilibrium and statistically significant (*p* < 0.05) differences in allele frequencies between the c.35delG homozygotes and the control samples are in bold

## Discussion

Distribution of the c.35delG carrier frequency in Russia is characterized by pronounced ethno-geographic specificity (Fig. 1). High c.35delG rates are typical for populations of Caucasian origin in north-western part of Russia (up to 7.5% in the Kaliningrad Oblast’ and 5.9% in the Leningrad Oblast’) [46]. The contemporary population of the Kaliningrad Oblast’ represented mainly by Russians (about 80%) was formed as a result of large-scale post-war (after 1945) migration from European regions of the former USSR. A high carrier frequency of c.35delG in rural areas of the Leningrad Oblast’ (5.9%) could probably be attributed to indigenous Finno-Ugric Vepsians, correlating with high c.35delG rates reported for other Finno-Ugrics: 4.4–4.5% in Estonians [12, 47], 5.7–6.2% in Mordvins [30, 48]. The highest carrier frequencies of c.35delG in the Caucasus are observed in Abkhazians (3.8%) [48] and Armenians (3.7%) [49]. Low carrier frequencies of c.35delG or its absence were observed among Turkic-speaking peoples of Volga-Ural region (Tatars, Bashkirs, Chuvashes), Siberia (Altaians, Tuvinians, Yakuts), Central Asia (Kazakhs, Uighurs, Uzbeks), and also in Mongolic-speaking Buryats (Siberia) [39–41, 48, 50]. In Siberia, the c.35delG carrier frequency in Russians varies from 4.1% in Western Siberia (this study) to 2.5% in Yakutia (Eastern Siberia) [41].

Haplotype (D13S141-rs3751385-D13S175-D13S1853) analysis of chromosome 13 in c.35delG homozygous deaf Russian patients from Siberia revealed the two most common haplotypes (126-T-c.35delG-105-202 and 124-T-c.35delG-105-202). It is interesting to compare the common c.35delG haplotypes identified in Siberia with available relevant data for other populations [19, 21, 23–26, 28, 29] (Table 3). Such comparisons are to some extent possible for the c.35delG haplotypes flanked by D13S141 and D13S175 (~ 124 kb). For these markers, the total share of two common c.35delG haplotypes in Siberia, 126-T-c.35delG-105 (56.3%, 27 of 48 chromosomes) and 124-T-c.35delG-105 (29.2%, 14 of 48 chromosomes), reaches 85.5% (Fig. 2B). Unfortunately, unified classification of the D13S141 and D13S175 alleles based on their size in nucleotides or number of dinucleotide (CA) repeats is absent due to different genotyping methods or simple numerical designations for alleles used in previous studies, making accurate comparative analysis hardly possible. Nonetheless, the 105 bp long allele of D13S175 was detected in the most common c.35delG haplotypes in the majority of studies while a broad variety of alleles was observed for D13S141. We were able to compare both allelic size in nucleotides (determined by fragment analysis) and numbers of CA repeats (detected by Sanger sequencing) in two most frequent D13S141 alleles in the c.35delG homozygotes from Siberia (analyzed in this study), Volga-Ural region of Russia (kindly provided by L. Dzhemileva [28]), and Belarus [29]. Fourteen CA repeats (CA_14_) were found in D13S141 alleles 126, 127, and 125 while thirteen CA repeats (CA_13_) were found in D13S141 alleles 124, 125, and 123 in DNA samples from Siberia, Belarus, and Volga-Ural region, respectively (Table 3). Thus, the identity of allelic composition of two most frequent haplotypes D13S141-c.35delG-D13S175 (~ 124 kb) was revealed in these geographically remote regions (Table 3). In addition, we assume that the conservative region of c.35delG haplotypes from Siberia may span longer being flanked by D13S141 and D13S1853 (~ 316 kb).

**Table 3:** The most common D13S141-c.35delG-D13S175 haplotypes in different populations

Common haplotypes D13S141-c.35delG-D13S175 (%)	Geographical region (Ethnicity)	References
126 ^a^-105 (56.3%), 124^b^-105 (29.2%)	Siberia, Russia (mostly Russians)	this study
125 ^a^-105 (67.9%)^c^, 123^b^-105 (12.5%)^c^	Volga-Ural region of Russia (mostly Russians and Tatars)	[28]
127 ^a^-105 (71.8%), 125^b^-105 (18.2%)	Belarus	[29]
3–4 (90%)^c^ 3–4 (100%)^c^	Palestine Israel	[19]
2–6 (34.5%)^c^, 3–5 (26.9%)^c^ 2–5 (42.9%)^c^, 3–5 (33.3%)^c^	Eastern Black Sea region, Turkey Other regions of Turkey	[21]
5 (127)-4 (105) (43%), 5 (127)-4 (105) / 4 (125)-4 (105) (18%)	Anatolia, Turkey	[23]
125–105 (83.3%)^c^, 123–105 (10.0%)^c^	Morocco	[24]
127–105 (61.5%)^c^, 125–105 (15.6%)^c^ 127–105 (60.3%)^c^, 125–105 (26.8%)^c^	Spain Greece	[26]

Allele destinations are taken from original sources. ^**a**^– fourteen CA repeats (CA_14_) was revealed by Sanger sequencing; ^**b**^– thirteen CA repeats (CA_13_) was revealed by Sanger sequencing; ^**c**^- frequencies of haplotypes were calculated on the basis of the data given in original sources [19, 21, 24, 26]

The contemporary Siberian population of Caucasian origin (mostly Russians) was formed as a result of multiple migration flows from the European part of Russia that started from the first settlement of Siberia by Russians at the end of sixteenth century [51]. Our rough dating of c.35delG expansion into Siberia (about 4800–8100 years ago) could be a reflection of complex processes of early formation stages of the modern European population (including the European part of Russia). These data do not contradict the earlier hypothesis about the c.35delG occurrence in the Middle East or the Mediterranean approximately 10,000–14,000 years ago followed by its spreading with migration flows across Europe [14, 17–27]. However, taking into account the current data on three ancient components in the origin of modern Europeans [52], it cannot be ruled out that c.35delG could also have independently originated among any ancient populations of North-West Europe or anywhere else.

## Conclusions

Distribution of the c.35delG carrier frequency in Russia is characterized by pronounced ethno-geographic specificity. High frequencies of c.35delG are observed in the populations living in north-western and central parts of Russia with a downward trend from west to east. The territory of Siberia can be assumed as the north-eastern geographic “end point” of the c.35delG prevalence in Eurasia. Comparative analysis of the c.35delG haplotypes supports the common origin of c.35delG in some regions of Russia (Siberia and Volga-Ural region) and in Eastern Europe (Belarus). A thorough study of the haplotypes associated with c.35delG in populations from different world regions could further elucidate its origin and age.

## Additional file

Additional file 1: **Table S1.** With detailed data on c.35delG carrier frequencies on the territory of Russia and in some countries of the former Soviet Union which were obtained from all available papers published up to 2018. This file also includes list of references for Table S1. (DOCX 42 kb)

## Abbreviations

*GJB2*: Gap junction protein, beta-2
SNP marker: Single nucleotide polymorphic marker
STR marker: Short tandem repeat marker
