# Supplementary material for: Updated carrier rates for c.35delG (GJB2) associated with hearing loss in Russia and common c.35delG haplotypes in Siberia
Source: BMC Med Genet. 2018 Aug 7;19:138. doi: 10.1186/s12881-018-0650-5 (PMC6081885; doi:10.1186/s12881-018-0650-5)
Supplement: Supplementary file 1 — Table S1. With detailed data on c.35delG carrier frequencies on the territory of Russia and in some countries of the former Soviet Union which were obtained from all available papers published up to 2018. This file also includes list of references for Table S1. (DOCX 42 kb) [file 12881_2018_650_MOESM1_ESM.docx]

**Table S1. The c.35delG carrier frequencies in the territory of the Russian Federation and in some countries of the former Soviet Union.**

| **Code** | **Regions** | **Characteristics of the samples** | | **Number of heterozygotes for c.35delG / sample size** | **Carrier frequency of c.35delG (%)** | **Reference**  *(see below Table S1)* |
| --- | --- | --- | --- | --- | --- | --- |
| **NORTHERN AND EASTERN EUROPE (*1-4*)** | | | | | | |
| ***1*** | Estonia | Estonians | 5/113 | | 4.4 | [11] |
|  | Estonia | Estonian neonates from general population | 45/996 | | 4.5 | [18] |
| ***2*** | Lithuania | Lithuanian population / Lithuanians | 1/98 | | 1.0 | [13] |
| ***3*** | Belarus | Belarusians | 6/97 | | 6.2 | [10] |
|  | Belarus | native residents of Belarus | 43/757 | | 5.7 | [9] |
|  | Belarus (Grodno Oblast’) | newborns from Grodno Oblast' | 8/234 | | 3.4 | [7] |
| ***4*** | Ukraine | Ukrainians | 3/90 | | 3.3 | [10] |
|  | Ukraine (Dnipropetrovsk and Zaporizhzhia regions) | newborns from Dnipropetrovsk and Zaporizhzhia regions | 8/193 | | 4.1 | [19] |
| **RUSSIAN FEDERATION (*5-31*)** | | | | | | |
| **North-Western part of Russia (*5-8*)** | | | | | | |
| ***5*** | Kaliningrad and Kaliningradskaya Oblast' | residents of Kaliningrad and Kaliningradskaya Oblast' | | 15/200 | 7.5 | [24] |
| ***6*** | Pskov and Pskovskaya Oblast' | residents of Pskov and Pskovskaya Oblast' | | 5/107 | 4.7 | [24] |
|  | Pskovskaya Oblast' | Russians | | 2/102 | 2.0 | [16] |
| ***7*** | St. Petersburg | residents of St. Petersburg | | 17/308 | 5.5 | [24] |
|  | Leningradskaya Oblast' *(Podporozhsii district)* | residents of Leningradskaya Oblast' *(Podporozhsii* *district*) | | 13/218 | 5.9 | [24] |
|  | Leningradskaya Oblast' *(Ivan-gorod)* | residents of Leningradskaya Oblast' *(Ivan-gorod)* | | 4/120 | 3.3 | [24] |
| ***8*** | Arkhangelsk and Arkhangelskaya Oblast' | residents of Arkhangelsk and Arkhangelskaya Oblast' | | 10/200 | 5.0 | [24] |
| **Central part of Russia (*9-10*)** | | | | | | |
| ***9*** | different regions of Russia | residents of different regions of Russia | | 20/391 | 5.1 | [5] |
|  | different regions of Russia | blood donors ( Russians) | | 38/1000 | 3.8 | [1] |
| ***10*** | Kirovskaya Oblast' | Russians | | 8/206 | 3.8 | [16] |
|  | | | | | | |
| **Table S1. (continued)** | | | | | | |
| **Volga-Ural region of Russia (*11-19*)** | | | | | | |
| ***11*** | Volga-Ural region of Russia | Komi | | 0/51 | 0 | [2] |
|  | Volga-Ural region of Russia | Komi-Permyaks | | 0/76 | 0 | [12] |
|  | Volga-Ural region of Russia | Komi-Permyaks | | 0/80 | 0 | [10] |
| ***12*** | Russia | Mari | | 5/194 | 2.6 | [2] |
|  | Volga-Ural region of Russia | Mari | | 1/49 | 2.0 | [12] |
|  | Mari El Republic | Mari | | 8/402 | 2.0 | [16, 20-23] |
| ***13*** | Volga-Ural region of Russia | Udmurts | | 2/61 | 3.3 | [12] |
|  | Republic of Udmurtia | Udmurts | | 2/434 | 0.7 | [21] |
|  | Republic of Udmurtia | Udmurts | | 3/592 | 0.5 | [16, 20, 22, 23] |
|  | Republic of Udmurtia | Udmurts | | 3/80 | 3.7 | [10] |
| ***14*** | Republic of Mordovia | Mordvins | | 4/70 | 5.7 | [12] |
|  | Republic of Mordovia | Mordvins | | 5/80 | 6.2 | [10] |
| ***15*** | Chuvash Republic | Chuvashes | | 4/154 | 2.6 | [2] |
|  | Volga-Ural region of Russia | Chuvashes | | 0/42 | 0 | [12] |
|  | Chuvash Republic | Chuvashes | | 3/400 | 1.5 | [21] |
|  | Chuvash Republic | Chuvashes | | 5/520 | 1.0 | [16, 20, 22, 23] |
|  | Chuvash Republic | Chuvashes | | 0/100 | 0 | [10] |
| ***16*** | Volga-Ural region of Russia | Russians | | 2/40 | 5.0 | [12] |
| ***17*** | Volga-Ural region of Russia | Tatars | | 1/85 | 1.2 | [12] |
|  | Republic of Tatarstan | Tatars | | 1/96 | 1.0 | [10] |
|  | Republic of Tatarstan | Tatars | | 6/252 | 2.6 | [23] |
| ***18*** | Republic of Bashkortostan | Bashkirs | | 2/55 | 3.6 | [2] |
|  | Republic of Bashkortostan | Bashkirs | | 1/400 | 0.3 | [10] |
|  | Republic of Bashkortostan | Bashkirs | | 0/208 | 0 | [12] |
|  | Republic of Bashkortostan | Bashkirs | | 2/396 | 0.5 | [16, 20, 22] |
| ***19*** | Ekaterinburg | Russians | | 2/92 | 2.2 | [10] |
|  |  |  | |  |  |  |
| **Table S1. (continued)** | | | | | | |
| **Siberia (*20-25*)** | | | | | | |
| ***20*** | **Novosibirsk, Western Siberia** | **residents of Novosibirsk (Russians)** | | **5/122** | **4.1** | **this study** |
| ***21*** | Republic of Altai, Southern Siberia | Altaians | | 0/130 | 0 | [15] |
|  | Republic of Altai, Southern Siberia | Altaians | | 0/230 | 0 | [10] |
| ***22*** | Republic of Tyva, Southern Siberia | Tuvinians | | 0/121 | 0 | [3] |
| ***23*** | Republic of Buryatia, South-Eastern Siberia | Buryats | | 0/261 | 0 | [14] |
| ***24*** | Republic of Sakha (Yakutia), Eastern Siberia | Yakuts | | 1/106 | 1.0 | [2] |
|  | Republic of Sakha (Yakutia), Eastern Siberia | Yakuts | | 1/247 | 0.4 | [4, 10] |
| ***25*** | Republic of Sakha (Yakutia), Eastern Siberia | Russians | | 2/80 | 2.5 | [4] |
| **South-Western part of Russia (*26-31*)** | | | | | | |
| ***26*** | Rostovskya Oblast' | residents of Rostovskya Oblast' (Russians) | | 19/660 | 2.9 | [16, 17, 20-22] |
| ***27*** | Karachay-Cherkess Republic, North Caucasus | Cherkessians | | 1/80 | 1.3 | [10] |
|  | Karachay-Cherkess Republic, North Caucasus | Cherkessians | | 2/102 | 2.0 | [14] |
| ***28*** | Karachay-Cherkess Republic, North Caucasus | Karachays | | 1/370 | 0.3 | [14] |
| ***29*** | Republic of Ingushetia, North Caucasus | Ingush | | 0/80 | 0 | [10] |
|  | Republic of Ingushetia, North Caucasus | Ingush | | 3/151 | 2.0 | [8] |
| ***30*** | Republic of Ingushetia, North Caucasus | Chechens | | 0/90 | 0 | [8] |
|  | Chechen Republic, North Caucasus | Chechens | | 1/147 | 0.7 | [8] |
| ***31*** | Republic of Dagestan, North Caucasus | Avars | | 0/60 | 0 | [10] |
| **SOUTH CAUCASUS (*32-33*)** | | | | | | |
| ***32*** | Abkhazia | Abkhazians | | 3/80 | 3.8 | [10] |
| ***33*** | Armenia | Armenians | | 8/219 | 3.7 | [6] |
| **CENTRAL ASIA (*34-36*)** | | | | | | |
| ***34*** | Uzbekistan | Uzbeks | | 0/60 | 0 | [10] |
| ***35*** | Kazakhstan | Kazakhs | | 2/240 | 0.8 | [10] |
| ***36*** | Kazakhstan | Uighurs | | 1/116 | 0.9 | [10] |

**References for Table S1**

1. Abramov DD, Belousova MV, Kadochnikova VV, Ragimov AA, Trofimov DYu. Carrier frequency of *GJB2* and *GALT* mutations associated with sensorineural hearing loss and galactosemia in the Russian population. Vestnik RGMU. 2016;6:20-4. [Article in Russian]
2. Anichkina A, Kulenich T, Zinchenko S, Shagina I, Polyakov A, Ginter E, Evgrafov O, Viktorova T, Khusnitdonova E On the origin and frequency of the 35delG allele in *GJB2*-linked deafness in Europe. Eur J Hum Genet. 2001;9:151.
3. Bady-Khoo MS, Bondar AA, Morozov IV, Zytsar MV, Mikhalskaya VYu, Skidanova OV, Barashkov NA, Mongush, RSh, Omzar OS, Tukar VM, et al. Study of hereditary forms of hearing loss in the Republic of Tyva. II. Evaluation of the mutational spectrum of the *GJB2* (Cx26) gene and its contribution to the etiology of hearing loss. Medizinskaya genetika. 2014;13:30-40. [Article in Russian]
4. Barashkov NA, Pshennikova VG, Posukh OL, Teryutin FM, Solovyev AV, Klarov LA, Romanov GP, Gotovtsev NN, Kozhevnikov AA, Kirillina EV, et al. Spectrum and Frequency of the *GJB2* gene pathogenic variants in a large cohort of patients with hearing impairment living in a subarctic region of Russia (the Sakha Republic). PLoS One. 2016; doi:10.1371/journal.pone.0156300.
5. Bliznets EA, Galkina VA, Matiushchenko GN, Kisina AG, Markova TG, Poliakov AV. Changes in the connexin 26 (*GJB2*) gene in Russian patients with hearing disorders: results of long-term molecular diagnostics of hereditary nonsyndromic deafness. Genetika. 2012;48:112-24. [Article in Russian]
6. Bliznetz EA, Sarkisian TF, Manoukyan TÀ, Bakhshinyan VV, Polyakov AV. *GJB2* caused hearing loss in Armenians. Medizinskaya genetika. 2012;5:23-28. [Article in Russian]
7. Bliznets EA, Marcul' DN, Khorov OG, Markova TG, Poliakov AV. The mutation spectrum of the *GJB2* gene in Belarussian patients with hearing loss. Results of pilot genetic screening of hearing impairment in newborns. Genetika. 2014;50:214-21. [Article in Russian]
8. Bliznetz EA, Lalayants MR, Markova TG, Balanovsky OP, Balanovska EV, Skhalyakho RA, Pocheshkhova EA, Nikitina NV, Voronin SV, Kudryashova EK, et al. Update of the *GJB2*/DFNB1 mutation spectrum in Russia: a founder Ingush mutation del(GJB2-D13S175) is the most frequent among other large deletions. J Hum Genet. 2017;62:789-95.
9. Danilenko N, Merkulava E, Siniauskaya M, Olejnik O, Levaya-Smaliak A, Kushniarevich A, Shymkevich A, Davydenko O. Spectrum of genetic changes in patients with non-syndromic hearing impairment and extremely high carrier frequency of 35delG *GJB2* mutation in Belarus. PLoS One. 2012; doi:10.1371/journal.pone.0036354.
10. Dzhemileva LU, Barashkov NA, Posukh OL, Khusainova RI, Akhmetova VL, Kutuev IA, Gilyazova IR, Tadinova VN, Fedorova SA, Khidiyatova IM, et al. Carrier frequency of *GJB2* gene mutations c.35delG, c.235delC and c.167delT among the populations of Eurasia. J Hum Genet. 2010;55:749-54.
11. Gasparini P, Rabionet R, Barbujani G, Melçhionda S, Petersen M, Brøndum-Nielsen K, Metspalu A, Oitmaa E, Pisano M, Fortina P, et al. High carrier frequency of the 35delG deafness mutation in European populations. Eur J Hum Genet. 2000;8:19-23.
12. Khidiiatova IM, Dzhemileva LU, Khabibulin RM, Khusnutdinova EK. Frequency of the 35delG mutation of the connexin 26 gene (*GJB2*) in patients with non-syndromic autosome-recessive deafness from Bashkortostan and in ethnic groups of the Volga-Ural region. Mol Biol (Mosk). 2002;36:438-41. [Article in Russian]
13. Mikstiene V, Jakaitiene A, Byckova J, Gradauskiene E, Preiksaitiene E, Burnyte B, Tumiene B, Matuleviciene A, Ambrozaityte L, Uktveryte I, et al. The high frequency of *GJB2* gene mutation c.313_326del14 suggests its possible origin in ancestors of Lithuanian population. BMC Genet. 2016;17:45.
14. Petrina NE, Bliznetz EA, Zinchenko RA, Makaov AKh.-M, Petrova NA, Vasilyeva TA, Chudakova LV, Petrin AN, Polyakov AV, Ginter E.K. The frequency of *GJB2* gene mutations in patients with hereditary non-syndromic sensoneural hearing loss in eight populations of Karachay-Cherkess Republic. Medizinskaya genetika. 2017;16:19-25. [Article in Russian]
15. Posukh O, Pallares-Ruiz N, Tadinova V, Osipova L, Claustres M, Roux AF. First molecular screening of deafness in the Altai Republic population. BMC Med Genet. 2005;6:12.
16. Sharonova EI, Osetrova AA, Zinchenko RA. Genetic causes of hereditary hearing impairment. Medizinskaya Genetika. 2008;**7**:23-8. [Article in Russian]
17. Shokarev RA, Amelina SS, Kriventsova NV, Elchinova GI, Khlebnikova OV, Tverskaya SM, Bliznetz EA, Polyakov AV, Zinchenko RA. Genetic-epidemiological and molecular study of hereditary deafness in Rostov province. Medizinskaya genetika. 2005;4:556-65. [Article in Russian]
18. Teek R, Kruustük K, Zordania R, Joost K, Reimand T, Möls T, Oitmaa E, Kahre T, Tõnisson N, Ounap K. Prevalence of c.35delG and p.M34T mutations in the *GJB2* gene in Estonia. Int J Pediatr Otorhinolaryngol. 2010;74:1007-12.
19. Veropotvelyan NP, Pogulyai YuS, Zhuravleva SA, Shutenko TV. Determination of total carrier frequency of mutation of 35delG gene in the connexin-26 gene among newborns of Dnipropetrovsk and Zaporizhzhia regions. Sovremennaya Pediatriya. 2015;1:130-4. [Article in Ukrainian]
20. Zinchenko SP, Kirillov AG, Abrukova AV, Sorokina TV, Sharonova EI, Khidiyatova IM, Dzhemileva LU, Shokarev RA, Bliznetz EA, Khusnutdinova EK, et al. Epidemiological study of hereditary hearing loss (nonsyndromal and syndromal) in Chuvashia Republic. Medizinskaya Genetika. 2007;6:19-29. [Article in Russian]
21. Zinchenko RA, El'chinova GI, Galkina VA, Kirillov AG, Abrukova AV, Petrova NV, Timkovskaya EE, Zinchenko SP, Amelina SS, et al. Genetic differentiation ethnic groups of Russia on genes of hereditary disorders. Medizinskaya Genetika. 2007;6:29-37. [Article in Russian]
22. Zinchenko RA, Murzabaeva SSh, Grinberg IaI, Galkina VA, Khlebnikova OV, Dadali EL, Fedotov VP, Khidiiatova IM, Khusnutdinova EK, Ginter EK. Genetic epidemiological study of Bashkortostan Republic: the diversity of monogenic hereditary diseases in five districts. Genetika. 2009;45:677-90. [Article in Russian]
23. Zinchenko RA, Galkina VA, Bessonova LA, Dadali EL, Khlebnikova OV, Mikhailova LK, Kadyshev VV, Petrin AN, Sharonova EI, Vafina ZI, et al. Medical-genetic study of the Tatarstan Republic. II. Diversity of hereditary diseases in three Districts of Republic Kazan-Tatar. Medizinskaya Genetika. 2012;9:31-40. [Article in Russian]
24. Zhuravskiy SG, Ivanov SA, Taraskina AE, Grinchik OV, Kurus AA. Prevalence of *GJB2* gene mutation 35delG among healthy population of Northwest Region of Russia. Med. Acad. Journ. 2009;9:41–5. [Article in Russian]
